# Supplementary material for: The interactome of intact mitochondria by cross-linking mass spectrometry provides evidence for coexisting respiratory supercomplexes
Source: Mol Cell Proteomics. 2017 Dec 8;17(2):216–32. doi: 10.1074/mcp.RA117.000470 (PMC5795388; doi:10.1074/mcp.RA117.000470)
Supplement: Supplemental Data [file supp_RA117.000470_133922_0_supp_23978_fzffwf.zip › spectra_annotation/mito_spectra_annotation/175-1-1-1-5-1.pdf]

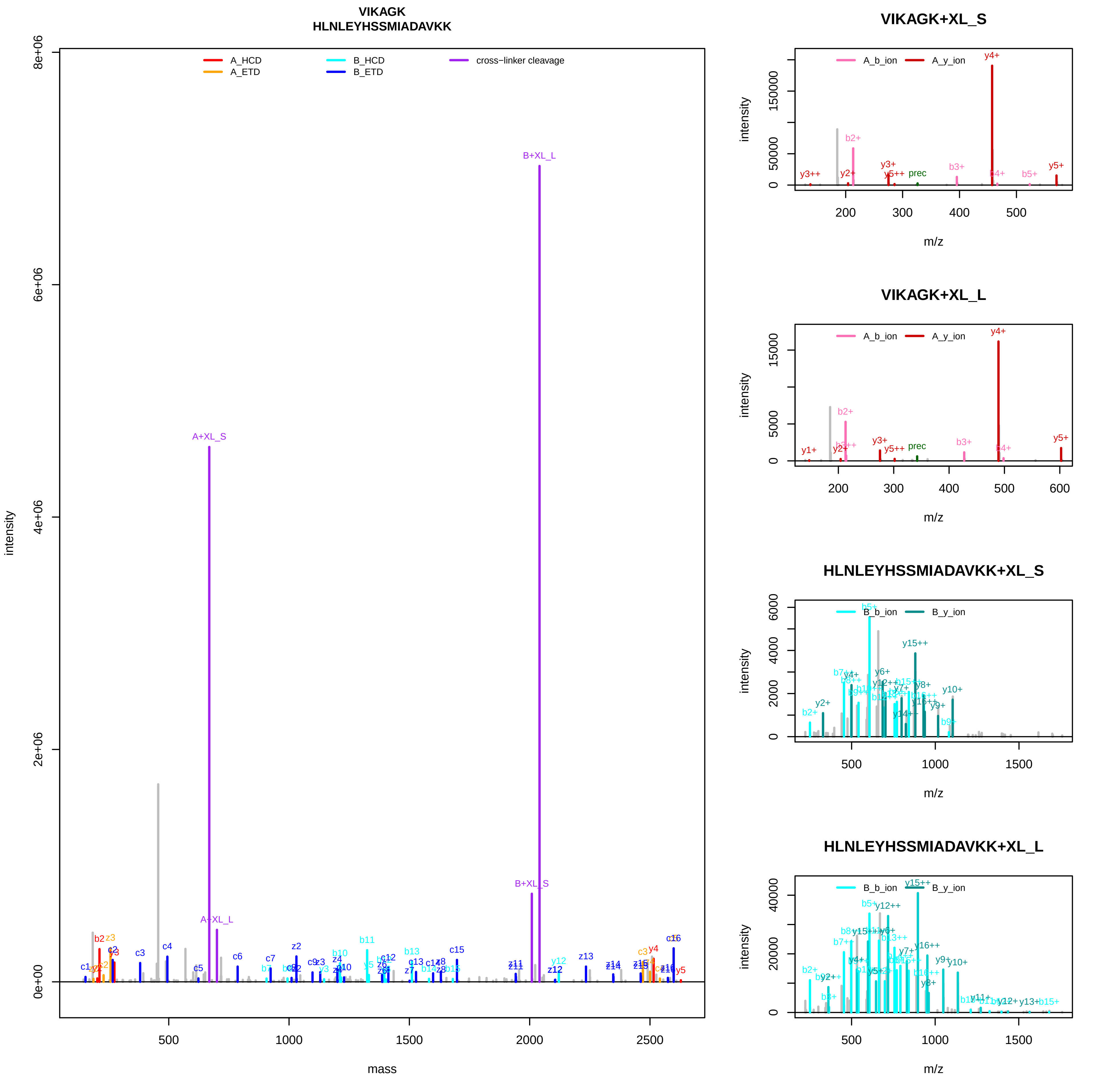
VIKAGK
HLNLEYHSSMIADAVKK
A_HCD
A_ETD
B_HCD
B_ETD
cross−linker cleavage
B+XL_L
A+XL_S
B+XL_S
A+XL_L
intensity
mass
0e+00
2e+06
4e+06
6e+06
8e+06
500
1000
1500
2000
2500
VIKAGK+XL_S
A_b_ion
A_y_ion
y4+
b2+
y3++
y2+
y3+
y5++
prec
b3+
b4+
b5+
y5+
intensity
m/z
0
50000
150000
200
300
400
500
VIKAGK+XL_L
A_b_ion
A_y_ion
y4+
b2+
y1+
y2+
y3+
y5++
prec
b3+
b4+
y5+
intensity
m/z
0
5000
15000
200
300
400
500
600
HLNLEYHSSMIADAVKK+XL_S
B_b_ion
B_y_ion
y15++
y10+
intensity
m/z
0
2000
4000
6000
500
1000
1500
HLNLEYHSSMIADAVKK+XL_L
B_b_ion
B_y_ion
y15++
intensity
m/z
0
20000
40000
500
1000
1500
